# Task-specific robot base pose optimization for robot-assisted surgeries

**DOI:** 10.3389/frobt.2022.899646

**Published:** 2022-12-02

**Authors:** Ashok M. Sundaram, Nikola Budjakoski, Julian Klodmann, Máximo A. Roa

**Affiliations:** Institute of Robotics and Mechatronics, German Aerospace Center (DLR), Wessling, Germany

**Keywords:** robotically assisted surgical systems, surgical robots, surgical setup planning, robot base pose optimization, capability maps, robotic arm

## Abstract

Preoperative planning and intra-operative system setup are crucial steps to successfully integrate robotically assisted surgical systems (RASS) into the operating room. Efficiency in terms of setup planning directly affects the overall procedural costs and increases acceptance of RASS by surgeons and clinical personnel. Due to the kinematic limitations of RASS, selecting an optimal robot base location and surgery access point for the patient is essential to avoid potentially critical complications due to reachability issues. To this end, this work proposes a novel versatile method for RASS setup and planning based on robot capability maps (CMAPs). CMAPs are a common tool to perform workspace analysis in robotics, as they are in general applicable to any robot kinematics. However, CMAPs have not been completely exploited so far for RASS setup and planning. By adapting global CMAPs to surgical procedure-specific tasks and constraints, a novel RASS capability map (RASSCMAP) is generated. Furthermore, RASSCMAPs can be derived to also comply with kinematic access constraints such as access points in laparoscopy. RASSCMAPs are versatile and applicable to any kind of surgical procedure; they can be used on the one hand for aiding in intra-operative experience-based system setup by visualizing online the robot’s capability to perform a task. On the other hand, they can be used to find the optimal setup by applying a multi-objective optimization based on a genetic algorithm preoperatively, which is then transfered to the operating room during system setup. To illustrate these applications, the method is evaluated in two different use cases, namely, pedicle screw placement in vertebral fixation procedures and general laparoscopy. The proposed RASSCMAPs help in increasing the overall clinical value of RASS by reducing system setup time and guaranteeing proper robot reachability to successfully perform the intended surgeries.

## 1 Introduction

Continuous advances in robotics exploit robotic arms beyond their conventional industrial use; in particular, sophisticated systems are nowadays deployed for performing various surgical procedures (Tylor 2006; Klodmann et al., 2021). Robotically assisted surgical systems (RASS) augment the experience and support the expertise of the physicians with dexterous instrumentation (Jelínek et al., 2015), high precision, and relentlessness. These systems have a master–follower architecture, that is, a teleoperation system, where the physician sends commands to the robot using an input device and receives a combination of haptic and visual feedback. High geometric accuracy is achieved with the use of robots, while the lead role and decision making on quantitative and unclear information is performed by the physician. The robot interface allows various sensor feedback modalities, such as vision and force, for the physician. This close and intuitive human–robot collaboration reduces the physical and cognitive burden for the physicians, at the same time benefiting the patient (Klodmann et al., 2021). RASS also enables the physician to perform surgeries in remote places and provide expertise without having to physically travel. In recent years, different RASS have been clinically tested and commercialized. They target various medical applications such as laparoscopy, microsurgery, intravascular, spinal, and cardiac procedures.

A preeminent step for a successful surgical intervention is the preoperative planning. It represents a systematic blueprint involving vital decisions such as the selection of access (incision) entry points in laparoscopy, which ultimately determine the outcome of the procedure. For instance, a poor choice of entry point can lead to limited reachability of the desired anatomical structure, causing unexpected complications. To avoid such complications in robot-assisted surgery, the setup of the robot’s base with respect to the access location and the choice of the access point to the patient must be done according to the access type and its constraints. The setup must guarantee that the robot can successfully perform the desired tasks during the surgical procedure in terms of dexterous manipulation capability. In addition, the overall technique must be reliable and time-efficient, as setup timing is one of the most criticized aspects of RASS by the surgeons (Taylor et al., 2016; Nezhat et al., 2006). Moreover, the setup pipeline must resolve the potential failures due to reachability and also be easy to use so that additional training of the clinical staff is not required. Finally, a proper solution should contribute to increasing the acceptance of RASS as a standard tool in surgical procedures.

Preoperative setup planning for surgical procedures strongly relies on clinical judgment and manuals, which are frequently combined with additional user interfaces (Pick et al., 2004). Research in optimal setup planning explores ideas such as virtual surgical simulations and multi-objective optimization for robot base and access point placement. Most of the proposed solutions that provide optimal workplace setups (Konietschke 2007) are time-consuming and rather complex approaches, or they are fast and simple, but lack optimization strategies (Lohmann and Konietschke 2012). In addition to preoperative setup planning, intra-operative system setup allows a physician to quickly modify and adapt the setup during surgery if required. Thus, a method to combine optimization-based preoperative setup planning and intra-operative setup experience is still missing.

This work contributes to filling this gap by exploiting the concept of robot capability maps (CMAPs) (Zacharias 2012; Porges et al., 2014; Porges et al., 2015) to aid preoperative setup optimization and intra-operative system setup. This type of workspace analysis allows efficient abstraction of the robot’s kinematic constraints and provides reachability information, e.g., the 6D poses reachable by the robot. Consequently, it can be used in applications involving task planning by utilizing and visualizing its discrete subspaces for particular tasks. Although CMAPs have been considered for preoperative setup planning, so far they lack useful information such as robot dexterity (Lohmann and Konietschke 2012) or consider limited task regions of interest (Zhang et al., 2021). Also, they are designed specifically for surgeries with access (incision) entry points, for instance, laparoscopy. In addition, the influence of the tool attached to the robot end-effector is also crucial but the tool orientation is not encoded so far in these approaches. Our work introduces a novel and general method that expands the well-established robot CMAP by considering the robot and tool kinematic constraints, the task constraints of the surgical procedure, and surgical access type constraints. This information is used for preoperative setup planning to optimize the robot base and access entry points placement. It can also be used online to aid the physician during intra-operative setup.

**FIGURE 1:**
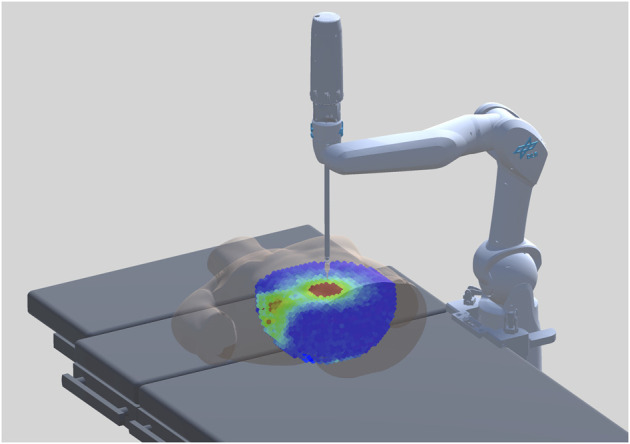
RASSCMAP generated for a laparoscopic procedure using the DLR MiroSurge system.

We propose an offline generation of a surgical procedure-specific RASS capability map (RASSCMAP), derived from the robot CMAP. Our method projects CMAP to discrete subspaces complying with the surgical access type constraints. For surgeries with access (incision) entry points, for instance, laparoscopy, we generate RASSCMAP for constrained access types (Figure 1). For surgeries without access entry points, for instance, spine surgery, we generate RASSCMAP for unconstrained access types. Furthermore, the RASSCMAP can be generated for medical robots of any kinematic type. Since fast online queries can be made to retrieve the map, it allows the physician to project the workspace of the robot over the task along with a capability metric based on the directional reachability information. Hence, this provides the ability to rapidly do intra-operative system setup and confirm the best poses for performing the surgical procedure.

The RASSCMAP can also be utilized to define a procedure-dependent optimization, where the procedure is a sequence of different tasks, such as preparation, resection, and reconstruction, represented as 6D task poses. Task trajectories are also discretized and represented as 6D task poses. These tasks are provided to a multi-objective optimization procedure to find the optimal placement for the robot base and access point location. We address the multi-objective, task-based optimization problem by utilizing Genetic Algorithms (David and Goldberg 1988), and we validate our results with the DLR’s surgical robotic platform MiroSurge (Seibold et al., 2018). In addition to introducing a novel basic concept for preoperative setup planning and intra-operative system setup, we verify the approach with two use cases: Pedicle screw placement in vertebral fixation of the spine, which exemplifies the concept for general unconstrained access types, and robot-assisted laparoscopy, which shows how to further incorporate access type constrains, that is, the access point for laparoscopic tools.

In Section 2, an overview of the state of the art is given. The concept of robot capability map and the methods for generating RASSCMAP for unconstrained and constrained access types are discussed in Section 3. In Section 4, task specification and setup optimization methods are presented. Section 5 describes the experiments and results for two use cases: 1) pedicle screw placement and 2) robot-assisted laparoscopy. A discussion of the results in comparison to the state of the art is provided in Section 6. Concluding remarks are given in Section 7.

## 2 Related work

A wide variety of techniques such as the use of preoperative manuals, virtual reality, multi-objective optimization, and kinematic analysis have been proposed for the preoperative setup of RASS. Among other surgery-specific medical decisions, the setup for RASS additionally includes deciding on appropriate access entry points and robot base placements. Early works for RASS setup rely on preoperative manuals based on clinical experience and multiple clinical trials, for example, cardiovascular endoscopy with the ZEUS surgical system (Austad et al., 2001), and robot-assisted prostate resections with the DaVinci surgical system (Pick et al., 2004). Similar methods based on clinical expertise are frequently deployed in practice due to their simplicity. The focus of these methods is more on the surgical target rather than the kinematic properties of the robot. Furthermore, virtual surgery simulations and augmented reality solutions based on medical imaging (Bauemschmitt et al., 2004) allow physicians to train, plan, corroborate, and adapt the intended surgery. The robotic manipulator can also be included in these surgical virtual environments (Hayashibe et al., 2005; Konietschke 2007). Even though virtual simulation-based methods offer clear insights into the surgery, they do not directly consider the robot’s kinematic capabilities. The extent to which the kinematic abilities of the robot are taken into account depends on the experience or knowledge of the user simulating the virtual environment.

Genetic Algorithm (GA) is frequently utilized to resolve highly constrained problems or multi-objective optimization processes in diverse areas such as pattern recognition (Fraser 1957), biological cells simulations (Bremermann 1958; Holland 1992), computer games (Bagley 1967), or aerodynamics shape optimization problems (Holst and Pulliam 2001). In robotics, GAs have been deployed for applications such as collision-free optimal motion planning (Watanabe and Izumi 1999), workplace optimization in cluttered environments (Makhal and Goins 2018; Bachmann et al. 2020), and surgical system setup planning (Konietschke 2007). In Tobergte et al. (2009), a GA was deployed to assist in preoperative optimal base and access points placement. The solution relies on a time-consuming and rather complex workflow. Constrained multi-objective optimization is used to assist physicians with access and robot joint configuration decisions in Adhami and Coste-Maniere (2003). The optimization considers generating a collision-free operation and reducing the inoperative organ motion. Here, the optimization goal is to find the preferred robot joint configuration rather than the robot base position.

For RASS setup planning, the robot reachability maps (Zacharias et al., 2007) have also been used. Early works on quantification of the robot’s ability to perform a task considered simple manipulability measurements based on the robot’s Jacobian matrix, which could for instance indicate closeness to singular configurations (Yoshikawa 1985). Dexterity computations using differential geometry were later introduced to support robot design optimization (Park and Brockett 1994). The initial attempts to describe the topology of the robotic systems started with explicit boundary extraction of the most distal points of the robot and encouraged research towards detailed workspace analysis (Gupta, 1986). However, the solutions were applicable to single grasp locations and did not account for directional data structures (Zacharias 2012). The reachability map proposed in Zacharias et al. (2007) encodes such directional structures. It also extends the workspace representation with a reachability index based on the robot’s forward kinematics. The index represents the dexterity of the robot at each 3D position. This index can be used for effective online planning for different manipulation applications (Porges et al., 2014; Porges et al., 2015). The capability map was initially applied for dexterity-based 3D path planning for grasping and manipulation tasks (Zacharias et al., 2010). The inverse values of a precomputed reachability map (Vahrenkamp et al., 2013) were also used to describe dexterities at different robot base placements. The work of Dong and Trinkle (2015) introduces a reachability map encoded with rotations as main criteria. It addresses online tool extensions, especially for tools with different kinematics, providing direct reuse of the map even without additional computations. Different map generation techniques are studied by Porges et al. (2014), analyzing mainly the generation time, memory footprint, and speed for online queries.

The direct use of the robot reachability map for RASS is not entirely suitable since it does not consider additional constraints such as robot operation through access points and additional degrees of freedom provided by the instrument attached to the end-effector. Therefore, new approaches emerged to consider some of these requirements for RASS. The concept of a trocar map (Lohmann and Konietschke 2012) reduces the total workspace of the robot to a surgery-constrained map computed in nearly real-time. The maps can be used to overlay and visually inspect desired task regions to aid in intra-operative setup. Although these trocar maps consider surgery-specific access constraints, they are an approximation and capture only 3D reachability information. The reachability index associated with each 3D position is also not considered. For precise setup planning, more accurate 6D reachability information is required. The work of Du et al. (2017) explores the kinematics of the robot in a virtually simulated surgical scenario to optimize the access entry points for a given robot base position. The algorithm uses two indices based on dexterity and collision avoidance. The time taken for the optimization in this approach is high due to the virtual simulation. Moreover, the possible number of access entry points for the patient is usually limited compared to the number of possible robot base positions. By optimizing only for the access entry point, the possible advantage of having a movable robot base is not exploited. Simultaneous optimization of robot base position and access entry point, as proposed in our method, allows for full utilization of the robot’s capability.

In the work of Zhang et al. (2021), the possible good regions for the robot base are computed based on reachability analysis and further refined by considering collisions. The reachability analysis is done while considering the access constraint and approximating the task space within the patient as a cone, where the apex of the cone is the access entry point and the base of the cone is the plane on which the robot needs to perform the task. The degrees of freedom of the instrument are not directly considered here. Therefore, the reachability analysis is purely based on the robotic arm. The physician can place the robot at a specific convenient position within the suggested region of high reachability. For the chosen robot base position, the initial robot joint configuration is also optimized. The complete task space of the robot operating through an access point within the patient is a hemisphere. By reducing this to a cone, only a specific region of the complete task space is computed. Although this might be enough to optimize for a specific task plane, it does not provide the ability to choose a robot base pose that also has a good overall reachable workspace. Considering a robot base position with a good overall reachable workspace is crucial for RASS since the physician might have to address some unplanned surgical complications outside the task region. The surgery-specific maps listed so far considered only surgeries with access entry points. A method to generate surgery-specific maps for procedures without access entry points is still open.

In summary, the following research gaps have been identified and addressed in this work:

• Consideration of different access constraints during map generation: surgeries with and without access entry points
• Task-independent map generation for the complete reachable workspace
• Full consideration of degrees of freedom of the instrument and the corresponding refinement of the map
• Task-specific simultaneous optimization of access entry point and robot base position
• Suitability for both preoperative and intra-operative setup planning

## 3 RASS capability map generation

The reachable workspace of a robotic arm is the set of all robot tool frame (TCP) poses that can be reached by the arm with some choice of joint angles. The reachable Cartesian workspace in $R^{3}$ can be analyzed by discretizing it as voxels with a desired resolution. Each Cartesian voxel can further have an associated rotational grid that discretizes *SO*(3). The reachability map, that is, the map that contains all reachable positions and orientations by the TCP, can be generated offline using this representation (Zacharias 2012; Porges et al., 2014). Within this map, a reachable 6D pose has an associated value of one stored in the map; the value is zero for an unreachable 6D pose. The map can be generated using forward or inverse kinematics (Porges et al., 2014). A memory-efficient data structure is used to store the map and enable fast online queries to check the reachability of a particular 6D pose.

**FIGURE 2:**
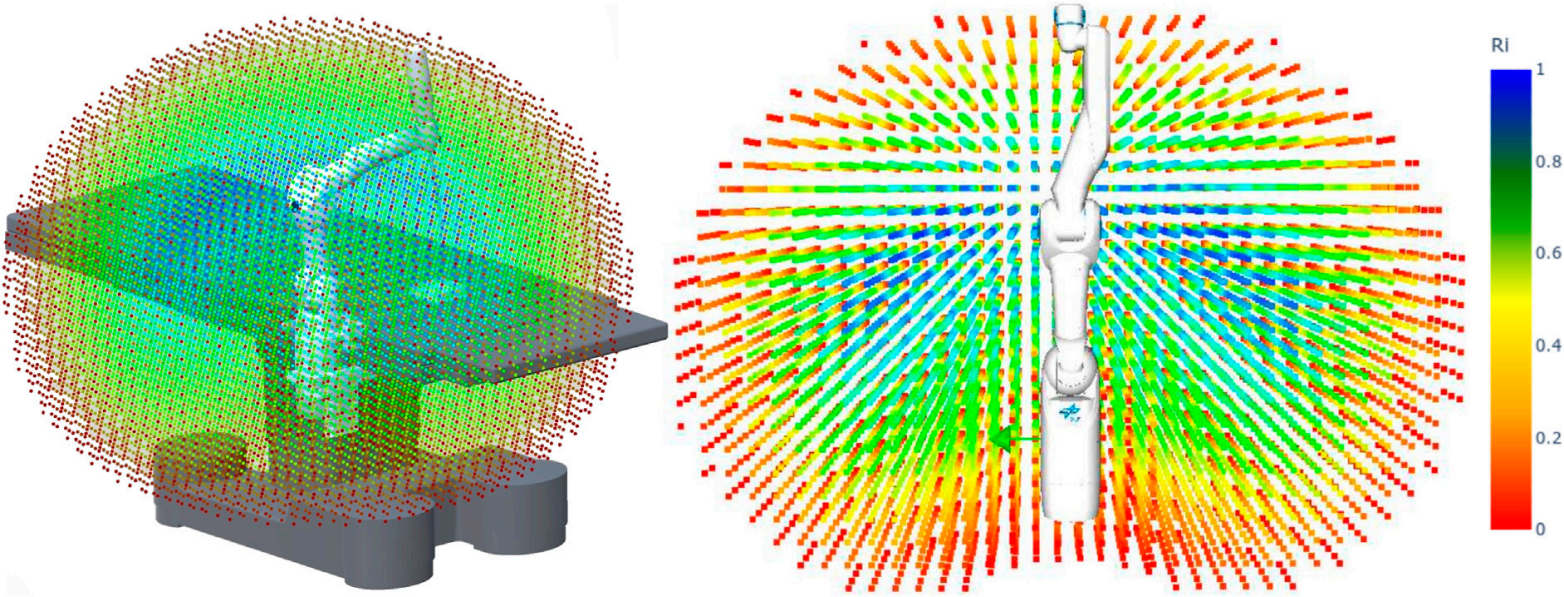
Capability map for the DLR MIRO robot arm. The reachability index is represented in HSV color scale, with red being zero (low dexterity regions) and blue being one (high dexterity regions).

In addition to the reachability map, a capability map can also be generated, which provides a quality index for each voxel or 3D position. This index, hereafter referred to as the reachability index, is computed as the number of reachable orientations in that voxel with respect to all the discretized orientations. The index provides then a value between zero and one, and represents the local dexterity of the robot in that voxel. A reachability index of one indicates that the robot can reach all the discretized orientations, thus it has high local dexterity in that voxel. Based on the reachability, the voxels can be color-coded (for instance on an HSV color scale, red being zero and blue being one) to allow a quick inspection of the robot’s workspace. As an example, Figure 2 shows the capability map for the DLR MIRO robot arm. With the help of reachability and capability maps, the robot base position can be optimized in order to achieve a successful task execution with high dexterity. Further details on reachability and capability map generation are provided in Porges et al. (2014).

The generation of the reachability and capability maps does not consider any constraints beyond the joint limits of the robotic arm. For a surgical task, additional constraints imposed by the access type are of prime importance. This modifies the actual robot’s reachability and capability for performing the intended tasks. The following sections discuss the RASSCMAP generation and utilization for unconstrained and constrained access types.

### 3.1 RASSCMAP for unconstrained access types

With RASSCMAP for unconstrained access types (for example, a spine surgery), the challenge lies in fitting the map as good as possible along the surface of the human limb for which the surgery is performed. This ensures reliable dexterity estimation to reach the entire human limb region on which the surgery is performed. In contrast, RASSCMAPs generated for constrained access types (for example, a laparoscopy) primarily consider the constraint occurring due to the access point.

**FIGURE 3:**
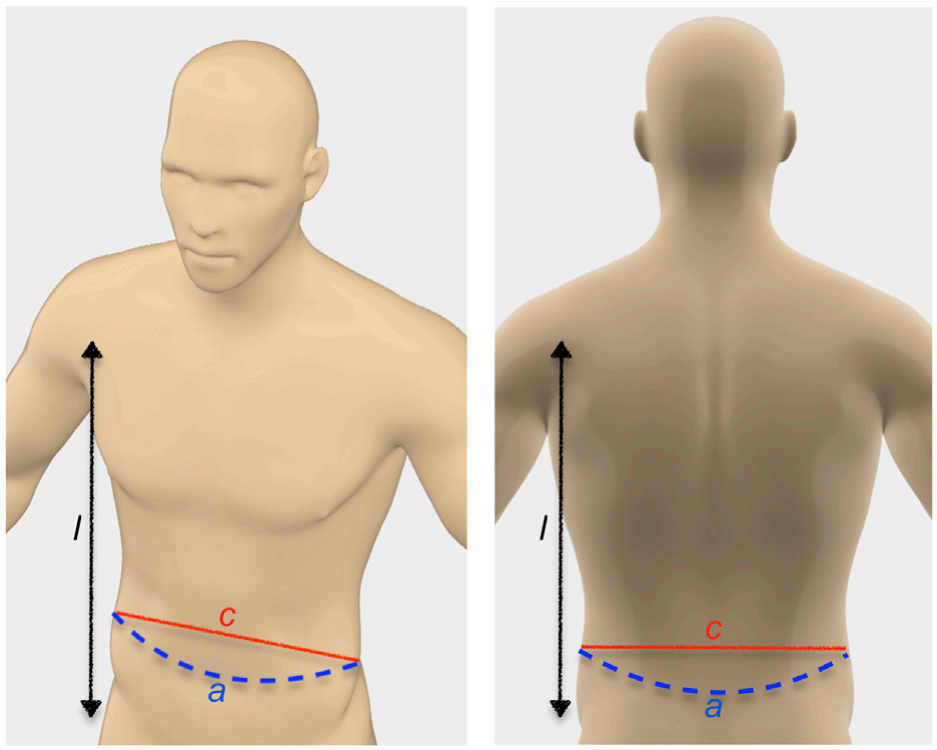
Parameters to approximate the patient’s limb and body surfaces.

To generate RASSCMAP, all human limbs are approximated as an elliptical cylinder by measuring their circumference, width, and length, as illustrated in Figure 3. For example, for surgeries in the abdomen region, the circumference *a* can be measured as the surface distance from one side to the other. The width *c* is defined then as the shortest distance from one side to the other, and the length *l* corresponds to the length of the abdomen region. If the surgeon prefers high robot dexterity in the complete abdomen and chest region, this can be added together to obtain the length *l*. Similar considerations can also be made for surgeries on the spine. Other body parts, such as the different sides of the leg, arm, and head can also be approximated by taking similar measurements. During the preoperative planning phase, these parameters need to be determined, either by manual measurements or automatically from preoperative images like CT and MRI scans. They are stored in a file as discussed in Section 4.1. For RASSCMAP generation, the required parameters are provided by parsing these files.

**FIGURE 4:**
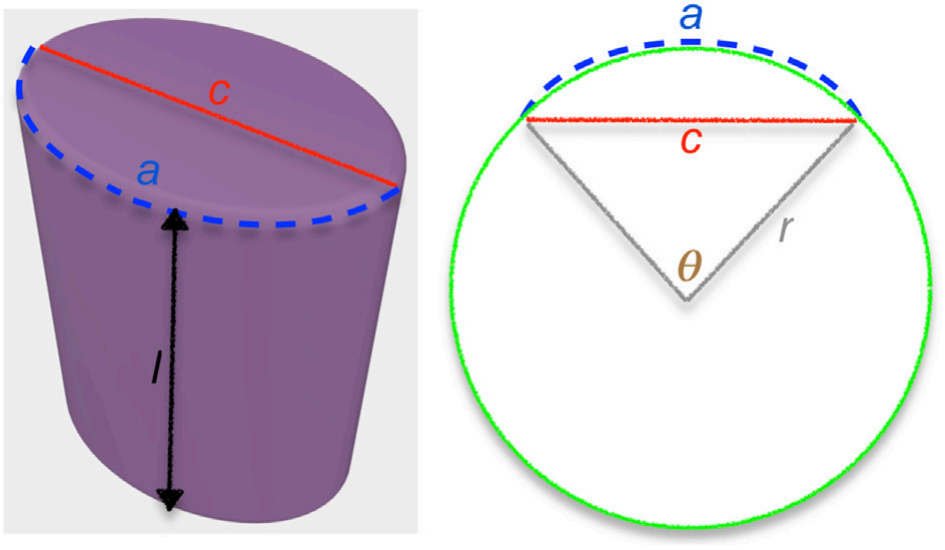
Approximation of the body part as an elliptical cylinder.

With these measurements, it is possible to approximate the geometry of the desired body part as an elliptical cylinder, using *a* as the arc length and the chord length *c* as the major axis of the ellipse (Figure 4). Using these values, the radius *r* of the circle in which this arc lies can be solved numerically as $c=2r\sin\left(\frac{a}{2r}\right)$ . Arc angle *θ* can be calculated as $\theta =\frac{a}{r}$ . The number of possible rotations, that is, the size *n* of the set Θ, is determined by the rotational discretization factor *d*_*r*_ as $n=\left\lfloor \frac{\theta }{d_{r}}\right\rfloor$ . The discretization factor is decided during the planning phase and made available through a YAML file. The set Θ consists of *n* linearly spaced rotations in the range Θ_1_ to Θ_*n*_.

$$\Theta =\left[\Theta_{1},\Theta_{2},\ldots ,\Theta_{n-1},\Theta_{n}\right],with\;\;\Theta_{1}=\frac{180-\theta }{2}\;\;and\;\;\Theta_{n}=\frac{180+\theta }{2}.$$ (1)

**FIGURE 5:**
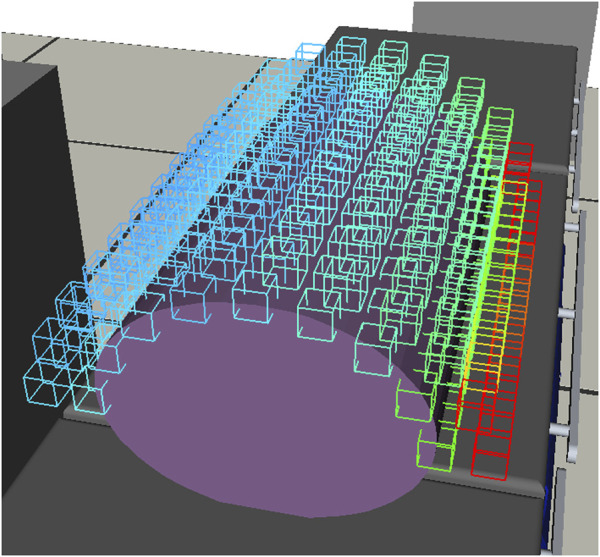
RASSCMAP generated for a spine surgery. The map fits the surface of the human body, which is approximated as an elliptical cylinder.

For each possible rotation Θ_i_ in Θ, the *x* and *y* position on the circle can be calculated as *x* = *r* cos(Θ_i_) and *y* = *r* sin(Θ_i_). For each (*x*, *y*) position, the *z* position is discretized from 0 to *l* in steps of *d*_*t*_ (translational discretization factor), as defined during the planning phase. By transforming these 3*D* points to the robot reference frame, the CMAP of the robot can be queried and saved to form the RASSCMAP around the surface of the patient’s area of interest. The depth of the RASSCMAP, that is, the number of layers in the map, is determined by the procedure to be conducted. For each layer, the radius of the circle is incremented by the voxel size *v*_*size*_ of the RASSCMAP. An example of RASSCMAP with two layers for a spinal vertebral fixation procedure is shown in Figure 5. The map fits the surface of the elliptical cylinder that approximates the geometry of the human.

### 3.2 RASSCMAP for constrained access types

The RASSCMAP for constrained access is derived from the robot’s CMAP while complying with the procedure kinematic constraints. The map is computed offline for all candidate access points in the CMAP and stored for further processing. In the following, we exemplify how such constraints can be incorporated in a laparoscopy use case. Laparoscopy is a procedure performed via multiple access points that allow the physician to examine and manipulate internal organs by introducing specialized surgical tools via dedicated trocar systems (Dankelman et al., 2011). An optimal choice of the robot’s base location and access points is crucial to avoid potentially critical complications during the procedure. An unplanned change of an access point would result in additional trauma, increased operational time, and additional costs.

**FIGURE 6:**
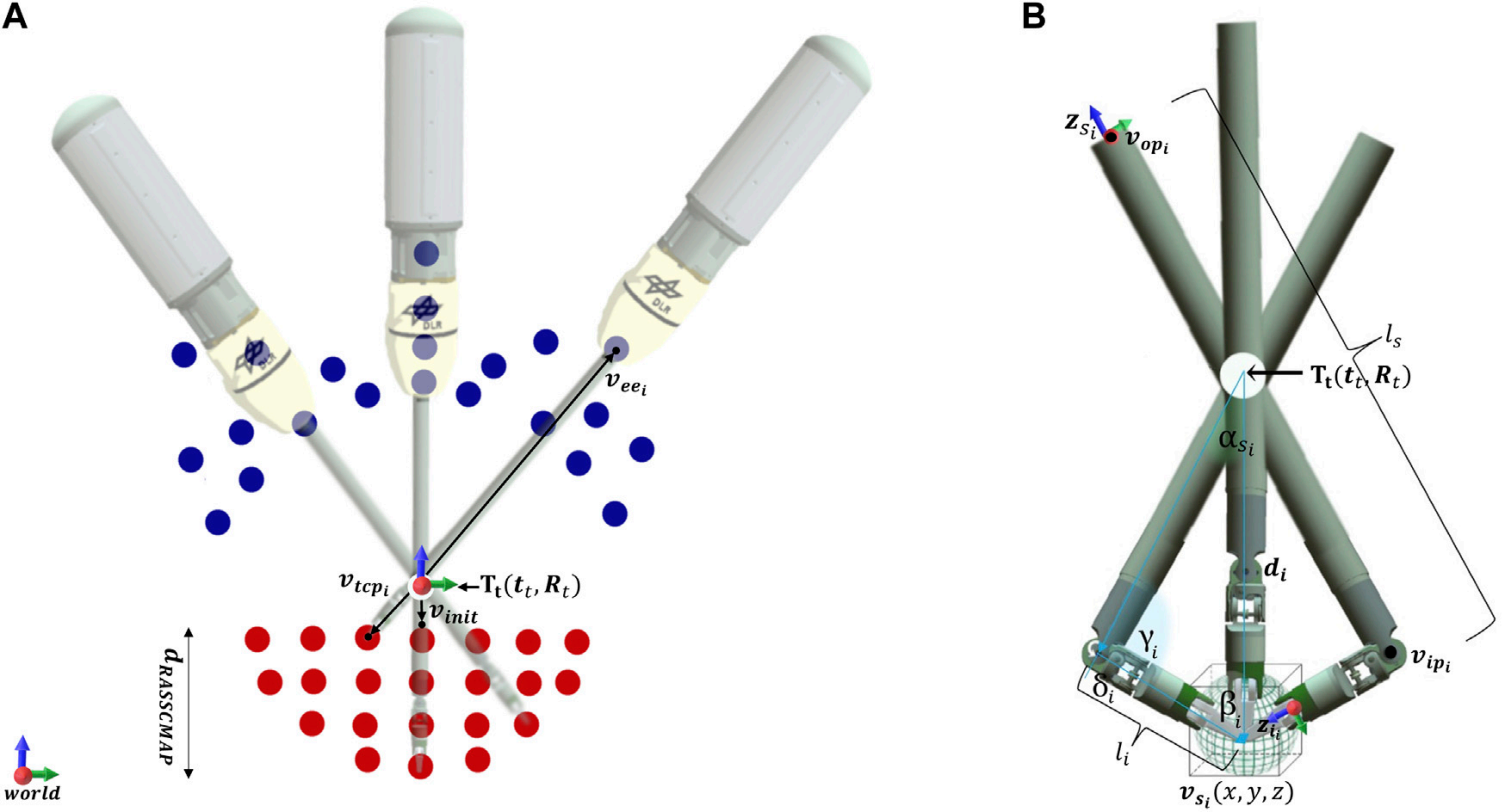
Computation of the RASSCMAP. **(A)** Three iterations of RASSCMAP shape generation process showing different instrument penetration depths and rotations around the entry access point (**T**_t_). **(B)** Three iterations of RASSCMAP enhancement with instrument rotation around voxel pivot point (***v**_s___i__*).

The function for generating the map is represented as *f*(*D*_*p*_, *T*_*p*_, *L*_*t*_), where *D*_*p*_ defines the discretization of the $R^{3}$ and *SO*(3) spaces in terms of voxel size and approach directions. *T*_*p*_ is a structure containing access point parameters such as the access point pose **T**_*t*_, the length of the trocar cannula, the depth of the map, and the rotational constraints **R**_*tc*_ for the maximum rotation around the remote center of motion along the *x* and *y* axis. The surgical tool length *L*_*t*_ consists of a shaft with length *l*_*s*_ and an instrument with length *l*_*i*_. These variables are inputs for the user YAML file in the preoperative planning as explained in Section 4.1 and further illustrated in Figure 6.

The creation of the RASSCMAP map follows an algorithm consisting of two parts. The first part computes the voxels that can be reached by the instrument with at least one orientation, while the joints of the instrument are kept in the initial pose, i.e., zero degrees. This results in a RASSCMAP with minimum capability. The second part takes into account the joint rotation of the instrument inside the patient around a voxel center point, resulting in the final RASSCMAP as a union of directional structures with instrument capability information. The access point, also known as the trocar point, is considered as a remote center of motion, and two vectors are used to search the existing voxels in the CMAP that correspond to a RASSCMAP voxel, as given in Figure 6.

The distance between the translational vector of the access point and the current exploration depth in the map, denoted as **v**_*init*_ is considered as a radius of a sphere that is constantly increasing until the algorithm reaches the desired depth of the RASSCMAP ( $d_{RASSCMAP}$ ). The center of the sphere is the trocar point, and the maximum allowed rotations for generating the RASSCMAP around the *x* and *y* axis are *α*_max_ = *β*_max_ = 90°. Thus, the xy plane cuts the spherical structure resulting in a map with a hemispherical shape. The search space is discretized within the allowed angles and they form a homogeneous transformation matrix **T**(*i*, *j*), used to transform the **v**_*init*_ in the 3D space. Once **v**_*init*_ penetrates beyond the trocar canula length, it becomes a vector inside the patient denoted as ${\text{v}}_{tcp_{i}}$ , and represents a potential candidate for the RASSCMAP. **v**_*ee_i_*_ is the vector outside the patient, representing a 3D point on the robot CMAP. The homogeneous transformation matrix $T_{\mathrm{RASSCMAP}}\left({\text{v}}_{tcp_{i}},R_{\mathrm{RASSCMAP}}\right)$ , will be considered as a valid entry in the map only if the query $T_{\mathrm{CMAP}}\left({\text{v}}_{ee_{i}},R_{\mathrm{CMAP}}\right)$ exists in the CMAP. The rotations **R**_*CMAP*_ and **R**_*RASSCMAP*_ define the orientation of the vector in the space, and they are computed with the Rodrigues’ rotation formula between two vectors with respect to the vertical axis in the world coordinate system. The current voxel coordinates with minimum capability in the RASSCMAP are stored as voxel pivot points in a multidimensional array (*v*_*s*_) and are further processed in the second part of the algorithm. The pseudocode is given in Algorithm 1.

Algorithm 1 RASSCMAP with Minimum Capability()

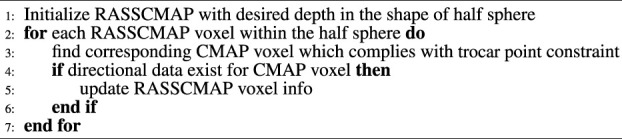

The second part of the algorithm takes into account the shaft rotations while the instrument is pivoting around the voxel center point. The RASSCMAP consists of voxels with comparably smaller size with respect to the CMAP voxels, as precision is a key factor in the surgical applications. Consequently, if the size of the voxels is small enough then the central point of the voxel is a good representation of all the points within the voxel. This part of the algorithm can be represented as *f*(*T*_*p*_, *l*_*i*_, *v*_*s*_) which stores the rotational data into a directional structure, directly defining the capability of the voxel. The algorithm checks each entry in the *v*_*s*_ array of size n and calculates the distance to the trocar point, given as follows:

$$d_{i}=\left\|t_{t}-v_{s_{i}}\right\|\;for\;i\in \left[0,n\right],$$ (2)

where ${\text{t}}_{t}$ is the translational part of the trocar access point ${\text{T}}_{t}$ . The maximum rotation of the shaft, while preserving the instrument tool tip on the center point as initially desired, is calculated based on the law of sines.

$$\gamma_{i}=180-\begin{array}{c} \delta_{i},\sin\left(\alpha_{s_{i}}\right)=\frac{l_{i}\cdot \sin\left(\gamma_{i}\right)}{d_{i}}, \end{array}$$ (3)

where δ in [0,90] represents the angle between the z axis of the shaft and the z axis of the instrument created due to the rotation of the instrument joints. For our application we use a pitch-yaw instrument with symmetric joint limits of ±90° (MICA, Seibold et al. (2005)). The procedure is repeated for both joint angles. Thus, the instrument pan and tilt angle define the *SO*(2) space, while the roll values are considered to correspond to the end-effector rotational joint limits of the robot, where any arbitrary rotation on the shaft roll axis can be achieved with the rotational joint of the robot end-effector. The maximum rotation pivot angle around the focus point for the instrument rotation is determined by the interior angles rule of triangles.

$$\beta_{i}=180-\left(\gamma_{i}+\alpha_{s_{i}}\right).$$ (4)

The current z-axis of the instrument (**z**_*i_i_*_) can be calculated from the current voxel pivot point (**v**_*s_i_*_) and the point inside the patient where the shaft ends (**v**_*ip_i_*_). The current z-axis of the shaft (**z**_*s*___*i*__) can be calculated from the point outside the patient where the shaft starts (**v**_*op_i_*_) and ${\text{t}}_{t}$ , which is the translational part of the trocar access point **T**_t_.

$$\begin{array}{c} \begin{array}{c} z_{i_{i}}=\frac{v_{s_{i}}-v_{ip_{i}}}{\left\|v_{s_{i}}-v_{ip_{i}}\right\|},\\ {\text{v}}_{op_{i}}=v_{ip_{i}}+l_{s}\frac{t_{t}-{\text{v}}_{ip_{i}}}{\left\|t_{t}-{\text{v}}_{ip_{i}}\right\|},\\ z_{s_{i}}=\frac{v_{op_{i}}-t_{t}}{\left\|v_{op_{i}}-t_{t}\right\|}. \end{array} \end{array}$$ (5)

The rotation for the sample in the CMAP and the sample in the RASSCMAP are calculated based on Rodrigues’ rotation formula, between the vertical axis of the world coordinate frame and the vectors $z_{i_{i}}$ , as well as the $z_{s_{i}}$ accordingly, as shown in Figure 6. If the calculated rotation is stored in the robot map, then the newly calculated orientation is added to the voxel of the RASSCMAP, implying that the orientation with such a configuration can be achieved inside the patient. The pseudocode for the second part of the algorithm is given in Algorithm 2. The final result is RASSCMAP with instrument capability as a union of directional structures, that can be generated offline and used online for fast queries.

Algorithm 2 Add RASSCMAP Orientations()

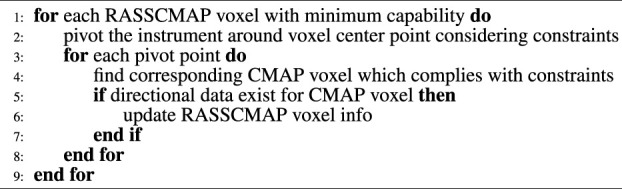

## 4 Task-specific setup optimization

The workflow for intra-operative system setup is straightforward with the use of RASSCMAPs. The RASSCMAPs can be generated offline for all possible base and access points and can be instantly visualized to help the physician decide on the surgical setup. If there is a need, RASSCMAPs can also be generated and visualized online. For a map with 0.05 m voxel size and 5° rotation discretization, the time taken is 8.7 s (Table 1).

**TABLE 1:** RASSCMAP total generation time (t) for different voxel size (v_*size*_) and rotation discretization (δ_R_), along with the time for the first (t_t_) and second part of the algorithm (t_R_), the total number of points (n), and the average capability of the full map.

*v*_*size*_(*m*)	*δ*_*R*_(°)	*t*_*t*_(*ms*)	*t*_*R*_(*ms*)	*t*(*ms*)	*n*	$\frac{1}{n}\cdot \sum_{i=0}^{n}r_{i}$
0.05	20	6,395	159	6,554	50	0.005985
0.05	15	6,407	291	6,698	50	0.0901428
0.05	10	6,309	611	6,920	50	0.110829
0.05	5	6,413	2,372	8,785	50	0.127257
0.05	1	6,410	58,311	64,721	50	0.1334486
0.04	20	8,898	446	9,344	124	0.0861012
0.04	15	8,677	770	9,447	124	0.127604
0.04	10	8,791	1,682	10,473	124	0.156786
0.04	5	7,001	6,512	13,513	124	0.180164
0.04	1	8,720	156,602	156,602	124	0.18064
0.03	20	11,824	1,065	12,889	308	0.0823169
0.03	15	11,559	1,841	13,400	308	0.0116705
0.03	10	9,372	3,626	12,998	308	0.146023
0.03	5	8,791	6,506	15,297	308	0.181786
0.03	1	11,585	376,478	388,063	308	0.190953
0.02	20	19,368	2,952	22,320	854	0.0663095
0.02	15	18,912	4,436	23,348	854	0.107189
0.02	10	19,459	10,975	30,434	854	0.125499
0.02	5	19,896	41,879	61,175	854	0.146192
0.02	1	19,572	380,278	399,850	854	0.168984
0.01	20	18,961	2,588	21,549	6,891	0.06760061
0.01	15	16,393	5,100	21,493	6,891	0.0687651
0.01	10	16,395	24,151	40,546	6,891	0.0978794
0.01	5	16,400	90,644	107,044	6,891	0.118518
0.01	1	16,285	1,027,435	1,043,720	6,891	0.133013

The workflow for preoperative task-specific setup optimization starts with gathering specific inputs related to the task, patient, and robot. This is done by the clinical expert as described in Section 4.1. Based on these inputs, the RASSCMAP (Section 3) is generated for all possible robot base and access point locations. Finally, a genetic algorithm is used for setup optimization (Section 4.2), to find a robot base and access point location that ensures reachability for all task poses and good dexterity in the surgery region.

### 4.1 Surgical task specification

In order to successfully optimize the robot base pose for a robot-assisted surgical task, different properties need to be considered. Specific information related to the patient, the surgical tasks, and the robot is required for this process. All these aspects are determined during the planning phase conducted before the actual surgery begins. In our implementation, human-readable YAML files are used to provide the data to the overall system. At runtime, the YAML files are parsed to obtain the necessary parameters for surgical procedure-specific RASSCMAP generation and task-specific robot base pose optimization.

The necessary patient information includes: a unique identifier for the patient, the position and orientation of the patient (or the position and orientation of a specific part of the patient’s body) on the operating room (OR) table with respect to the world coordinate system, and a suitable voxel size and map depth of the RASSCMAPs to be generated. The selection of voxel size and map depth depends on aspects such as the instrument mounted on the end-effector and the intended surgical tasks. Furthermore, depending on the surgery access type, other parameters related to the patient are also required. These parameters are used to generate the appropriate surgery-specific RASSCMAPs. For surgeries with constrained access type (for instance, laparoscopy), the necessary info includes the position and orientation of various possible access entry points for the trocar system determined with respect to the world coordinate system. In the case of surgeries with unconstrained access type (for instance, spine surgery), the necessary info includes the geometry of the patient, i.e., the width, half-circumference and length of the patient’s body part. The measurements are made (as described in Section 3.1) for the specific body part of the patient for which the surgery needs to be performed. For example, to perform a surgery on the spine, the width and circumference can be measured in the hip region, and the length can be determined by the number of vertebrae involved in the surgery. An example of the patient YAML file is shown in Listing 1.

Listing 1 YAML file listing the patient info.

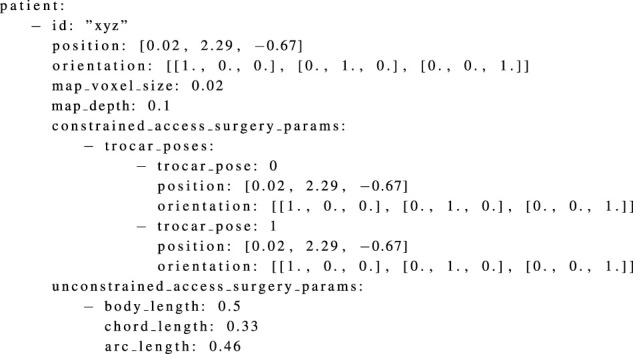

**FIGURE 7:**
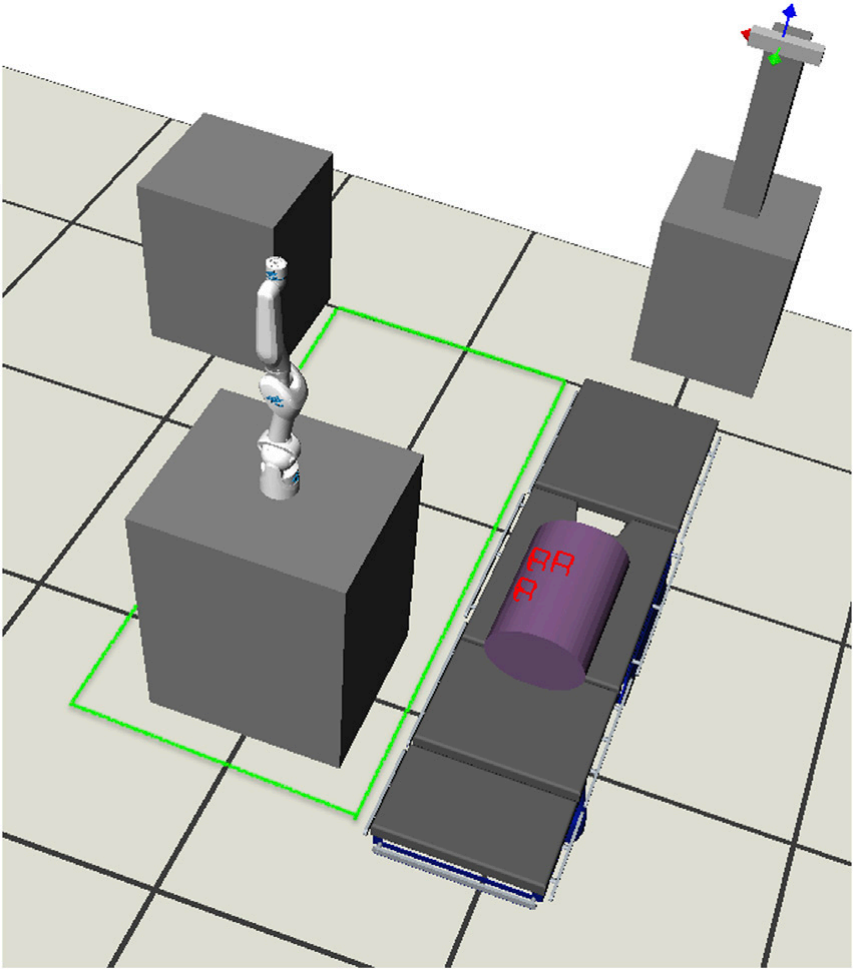
Pedicle screw placement positions (highlighted in red on the elliptical cylinder approximation of the patient’s spine region) and free region for the robot base (highlighted in green on the floor).

The task YAML file includes a list of different task poses of interest. The surgeon determines these poses during the preoperative planning phase. The robot base pose is optimized such that these task poses are reachable and the best possible dexterity is available at these poses. An example of the task YAML file is shown in Listing 2. The necessary information in the robot YAML file includes the name of the robot, the path where the robot CMAP is stored, and the translational and rotational discretization factors to be considered for the given range of possible robot base poses. The robot YAML also includes the list of possible base poses where the robot can be placed. The possible base poses are captured as a range. The minimum and maximum values for each x, y, and z translation as well as for rotation are given. If required, multiple possible base pose ranges can be used. This is helpful, for example, to provide the possible base pose range on each side of the OR table. In Figure 7, the free region for the base (highlighted in green) and the world coordinate system are shown. An example of the robot YAML file is shown in Listing 3. The position and orientation parameters in all YAML files are captured with respect to the world coordinate system. If a tracking system is available in the operating room, this could also act as the origin of the world coordinate system. It also makes it easy for the surgeon to move and measure various aspects directly through the tracking system user interface. The process of acquiring and saving all the parameters in the YAML file can also be automated by an information acquisition system^1^.

Listing 2 YAML file listing the task information.

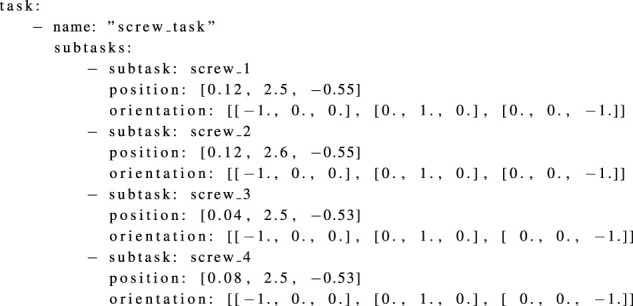

Listing 3 YAML file listing the robot information.

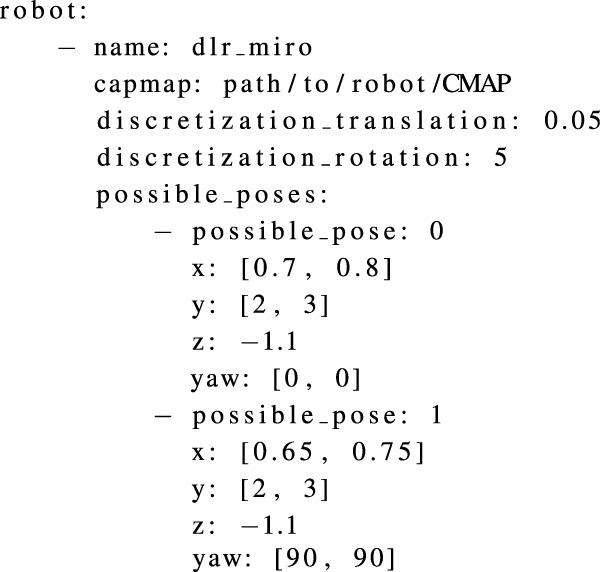

### 4.2 Setup optimization

For preoperative surgical setup optimization, we employ a genetic algorithm (GA). GA is a stochastic approach mostly deployed for constrained non-linear optimization problems with large search spaces. It follows the idea of natural evolution and reproduction. A diverse population of individuals is initialized and only the fittest survive for the next generation. The next generation population is updated by evolutionary operators such as selection, crossover, and mutation. GA searches an overpopulation of individuals rather than evaluating single instances and thereby avoiding local minimum. Moreover, discontinuous and non-differentiable objective functions can also be considered, which makes it suitable for discrete optimization problems. Detailed theoretical background about GA is given in David and Goldberg (1988). GA has been successfully used in the past for robotic work cell layout optimization including base repositioning and considering various non-linear constraints (Bachmann et al., 2020). Due to all these properties of GA, it is a suitable tool for surgical setup planning applications as well.

For preoperative surgical setup optimization, we consider the discrete problem of finding the best-suited robot base position and access entry points for the task within the allowed boundary constraints. The RASSCMAP, described in Section 3, provides a way to assess all possible solutions quantitatively. The RASSCMAP is a non-linear mapping of the robot’s kinematic and surgery-related access constraints. Depending on the use case, low discretization factors might have to be considered to have a higher sampling of possible base poses in the defined range and therefore find a more optimal solution.

For surgical setup optimization, the cost function used by GA to evaluate each base and access location is given by

$$f_{g}=\min\left(S_{r}\right)\cdot \overset{m}{\underset{i=1}{\prod }}c_{\left(t,i\right)}\cdot c_{s}.$$ (6)

For *m* task poses, reachability *S*_*r*_ = {*r*_(*t*,1)_, …, *r*_(*t*,*m*)_}, reachability index {*c*_(*t*,1)_, …, *c*_(*t*,*m*)_}, and the average capability of the surgery-specific RASSCMAP *c*_*s*_ are considered for cost computation. Reachability is a binary value associated with each pose, where one and zero represent a reachable or unreachable pose, respectively. If one of the tasks poses is not reachable, then the cost becomes zero, as this is the most important constraint to be met. Reachability index is the quality metric of a robot’s local dexterity in the voxel containing the pose in the range of zero to one, where one is the maximum dexterity. The average capability for the RASSCMAP is determined by adding the reachability index of all voxels on the map and dividing it by the number of voxels on the map.

## 5 Experiments and results

In this section, we outline two use cases considered for evaluating the proposed RASSCMAP-based setup planning. The first one explores the optimization of the robot base pose for a robot mounted on a mobile platform in order to perform a pedicle screw placement surgery. The second use case explores robot-assisted laparoscopy. Here, we first optimize the access point for a fixed robot base pose. Later, we perform the simultaneous optimization of robot base pose and access point.

### 5.1 Pedicle screw placement

Performing pedicle screw placement with robot assistance has numerous advantages, including increased accuracy, reduced hospital stay, and reproducibility. For this use case, we consider the robot arm mounted on a mobile platform. The height of the robot is fixed and it can move freely in the OR floor. The physician determines the possible range of base positions available for the mobile robotic platform around the OR table. The physician also determines various positions at which the screw insertion needs to be done, and provides all the required inputs as described in Section 4.1. Figure 7 shows the free region where the robot base can be located, highlighted in green on the floor, and the three screw placement positions highlighted in red on the elliptical cylinder that approximates the patient’s geometry.

The RASSCAMP for unconstrained access type is generated offline after all inputs are provided to the setup planning module. Due to the placement of multiple pedicle screws, while the robot’s base remains in a fixed location (to avoid redoing the registration), no additional constraints like in robot-assisted laparoscopy need to be considered. Therefore, only the robot base pose needs to be optimized particularly for the planned screw poses and for the considered spine region. The free region for the base is discretized with 5 cm for translations and 5° for rotations. By doing so, for this specific example there are 800 possible base poses in the green region. If required, low discretization factors and other free regions for the base (for instance, the right side of the OR table) can also be considered as per the physician’s opinion. This will increase the total number of possible base poses, and the time taken to generate the maps will also increase accordingly. The time taken to generate RASSCMAP with 100 voxels per map is 20 milliseconds. The total time for generating RASSCMAP for all 800 base poses is 16 s. The computations were done on a Linux PC with an Intel Xeon E5-1630 v4 CPU at 3.70 GHz.

**FIGURE 8:**
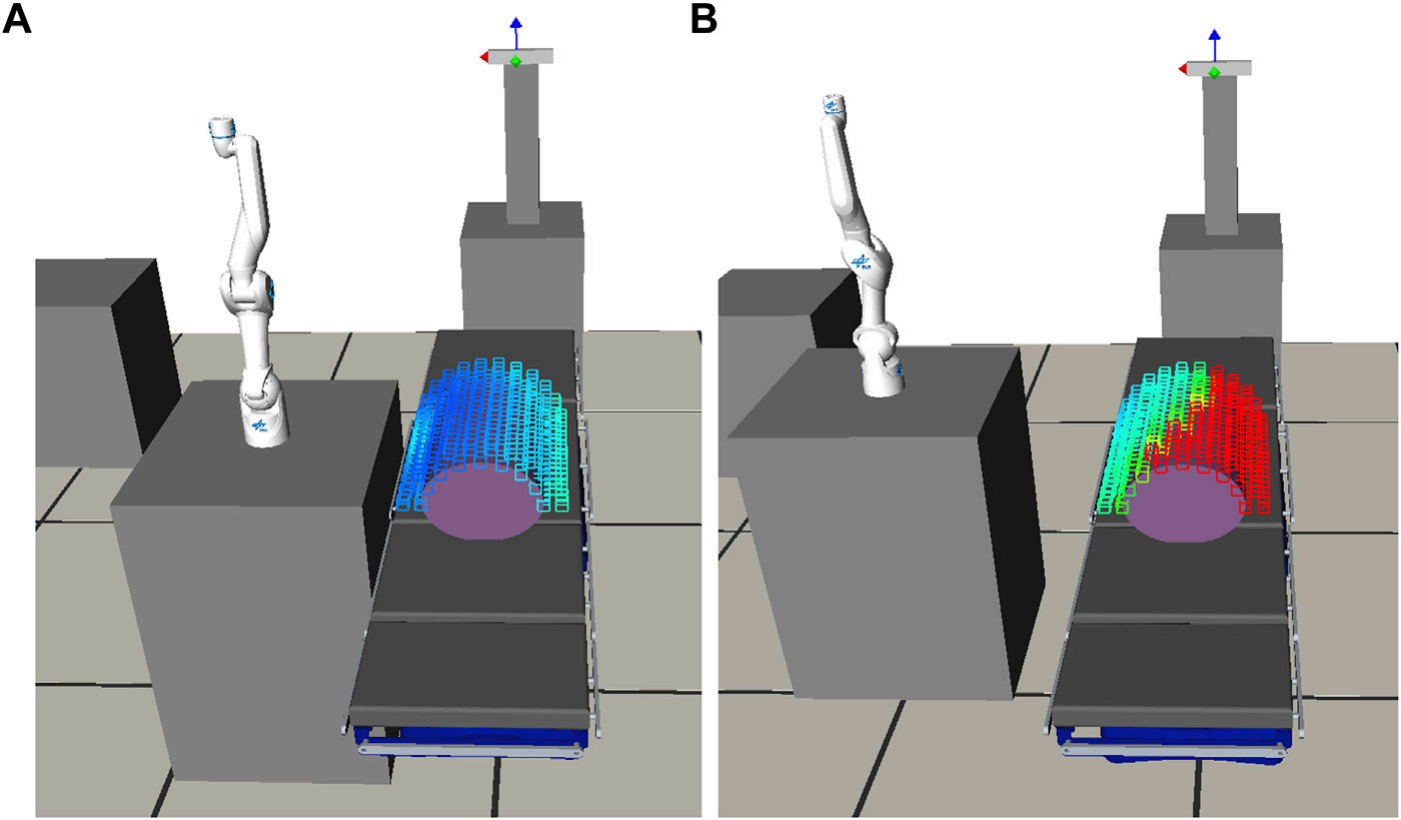
Robot base location for pedicle screw placement. **(A)** Best base pose along with RASSCMAP for this task. **(B)** Worst base pose along with RASSCMAP for this task. Note for instance the large unreachable region on the right side of the patient (in red).

The base pose is optimized so that the screw poses and the complete spine region are reachable with high dexterity. The time taken for optimization with the proposed cost for the genetic algorithm is in the range of 0.08–0.2 s. More details on optimization are provided in Section 4.2. The best base pose for this task is shown in Figure 8A. For comparison, by considering the additive inverse of the cost, the worst base pose for this task is also shown in Figure 8B.

### 5.2 Robot-assisted laparoscopy

**FIGURE 9:**
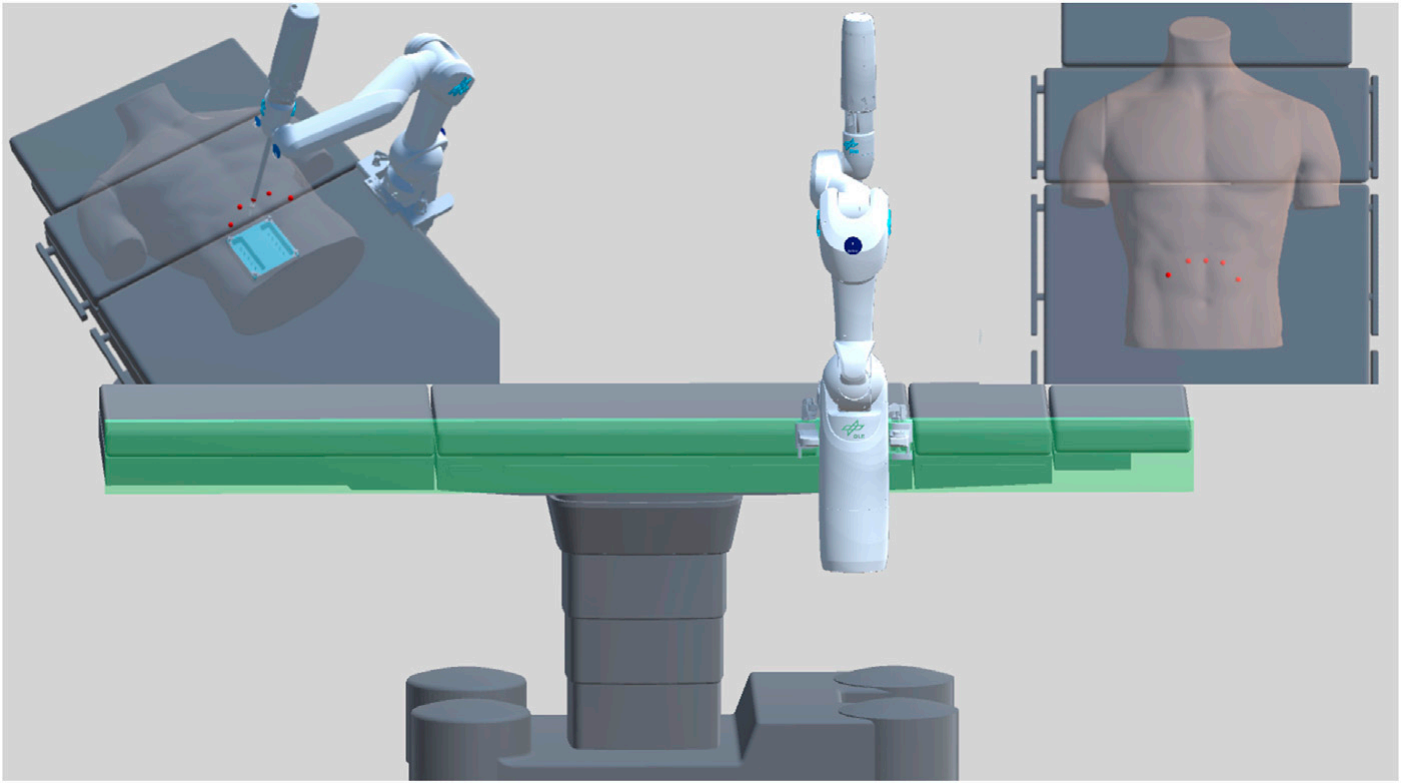
Robot-assisted laparoscopy. Area for possible robot base placement positions (highlighted in green), access point placement (highlighted in red), and the task as a desired area of motion on the surgical training pad (highlighted in blue).

The concept of laparoscopy replaces the conventional approach of open surgery whenever possible since it reduces the trauma, postoperative hospital stay, and provides better convalescence. For the use case of surgically assisted laparoscopy, modular kinematic manipulators can be mounted on an OR table with side rails, thus allowing translation of the base pose for proper base positioning along the *y*-axis in order to successfully execute the task. The range of the possible base poses and the desired access points are defined in the preoperative planning as described in Section 4.1. Figure 9 illustrates the OR table with one robot. The side rail on the OR table is highlighted in green.

The RASSCMAPs are precomputed offline for each access point and stored in a RASSCMAP database with a unique identification key, so they can be easily accessed. The computation times for different discretization parameters are given in Table 1. For the robot-assisted laparoscopy use case, two optimization problems are considered. First, optimizing the access points for a fixed base pose, and second, optimizing both the access points and the robot base pose simultaneously. To demonstrate the first part, we define five possible access points and tasks in the form of a cubical region on the top of the training pad where the robot should operate, resulting in 200 task poses. For the second part, in addition to the five access points, we utilize translation discretization of 5 cm along the *y*-axis (rail on which the robot is mounted), where the length of the rail is 1.2 m. The defined parameters are only an example consideration and can be adjusted according to the desired system and application.

#### 5.2.1 Optimization of multiple access points and a fixed base pose

**FIGURE 10:**
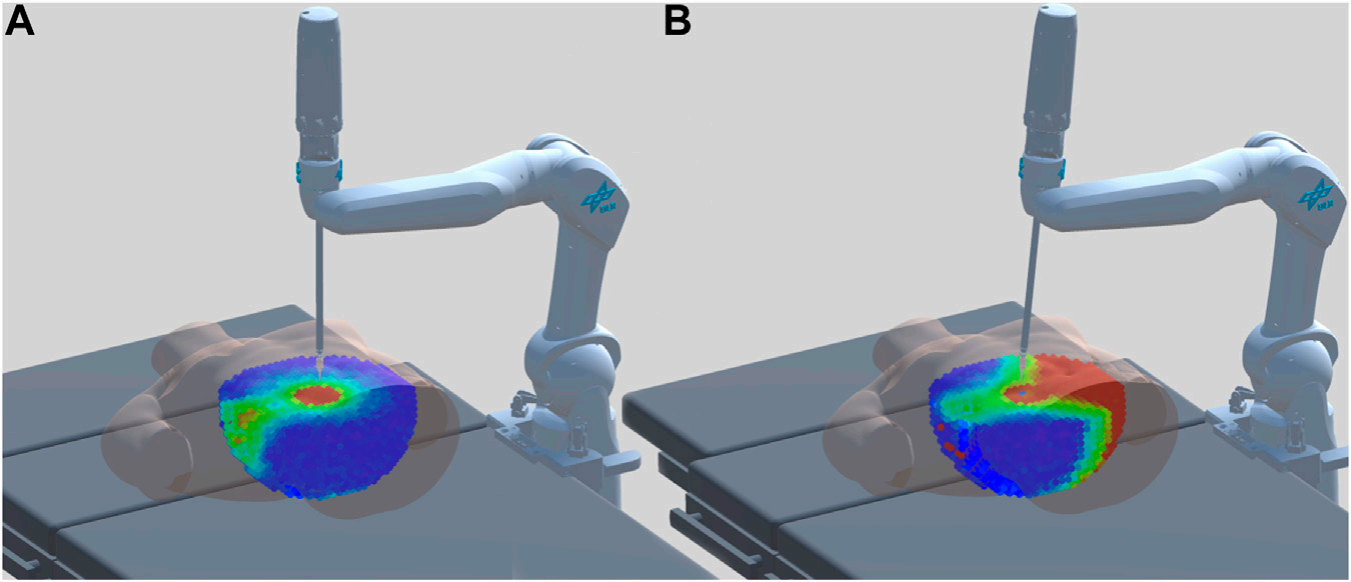
Visualization of the derived RASSCMAPs for constrained procedures for two different access points and fixed base pose. **(A)** Best-suited access point. **(B)** Worst-suited access point.

For this type of optimization, we consider a single fixed base pose and optimize for five different access points. The algorithm retrieves the RASSCMAP for the particular access point from the database and calculates the cost function as described in Section 4.2. This type of optimization can be used when the robot cannot be moved on the rail of the operating table due to the size of the OR surgery-specific constraints. The total time to find the optimal access point among the 5 possibilities for 200 task poses is 0.33 s. The computation is performed on a Linux Machine with Intel(R) Core(TM) i7-10700 CPU at 2.90 GHz. The visualization of the solution is given in Figure 10.

#### 5.2.2 Optimization of multiple access points and multiple base poses

For this type of optimization, we consider 25 different robot base poses along the rail and five different access points. The algorithm finds the corresponding access point from the RASSCMAP database for every robot base pose and calculates the cost as described in Section 4.2. The total time to optimize the full setup for a single robot that can move along the rails with 5 cm discretization for a range of 1.2 m with 5 access points and 200 task poses is 4.92 s. The computation is performed on a Linux Machine with Intel(R) Core(TM) i7-10700 CPU at 2.90 GHz.

## 6 Discussion

**FIGURE 11:**
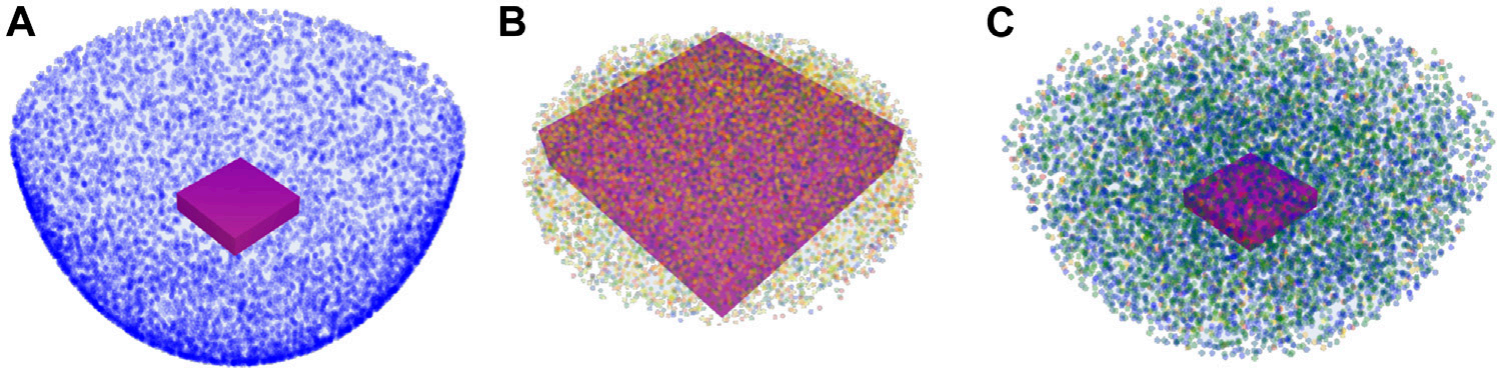
Illustrative comparison between the surgical capability maps proposed in the work of Lohmann and Konietschke (2012), Zhang et al. (2021), and RASSCMAP. The task poses are considered within the surgical training pad highlighted as a purple cuboid. **(A)** Lohmann and Konietschke (2012) represented the outline of the reachable workspace independent of the task. The map does not encode any dexterity data, and blue voxels represent reachable voxels. **(B)** Zhang et al. (2021) used a task-based map, where different colors imply different manipulability indexes of the robot end-effector. However, it does not encode the orientation of the surgical instrument. **(C)** RASSCMAP where different colors indicate different manipulability indices, while also considering the orientation of the surgical instrument. The map is task independent and provides the complete reachable workspace for the system.

Setup planning for RASS is a challenging task, as the proposed solution must be fast, accurate, and straightforward to use. This work introduced RASSCMAP, which describes the workspace of any robot in constrained and unconstrained surgical procedures. RASSCMAP is derived offline from the general CMAP of the robot and stored in a database that can be easily accessed during online queries. RASSCMAP for constrained access entry points is approximated by a hemisphere (Figure 11C). RASSCMAP for unconstrained access entry points is approximated by an elliptical cylinder. The robot CMAP is constrained as per the access type, and further refined by considering the degrees of freedom of the instrument attached to the end-effector. RASSCMAP represents 6D reachability information and includes a dexterity metric associated with each 3D position of the reachable workspace. This information allows direct comparison of the maps in the database based on their capability in the region of interest and the general coverage of the surgical site.

Some previous works have used information related to the robot’s capability in different ways. The approach presented in Lohmann and Konietschke (2012) generates a reduced workspace for the robot for constrained surgical procedures, based on the entry access point (Figure 11A). The solution neglects the degrees of freedom of the instrument and considers 3D positions on the border of the surgical workspace, validated via inverse kinematics. Hence, the surgical map represents the outline of the reachable workspace within the patient, without any additional dexterity information, which is particularly important for planning tasks where a high dexterity during surgery is required. Thus, the reduced robot workspace in the form of a surgical map can only determine if the desired task region is located within the interior part of its structure. The overall time for computing such a map is below 1 s.

A surgical map for constrained surgical procedures is also presented in Zhang et al. (2021). The reachable workspace for RASS is approximated by a set of cones of different heights whose apex is at the access entry point and the base is the plane on which some of the task poses lie. The map has a structure of a cylinder created by stacking the base of all cones (Figure 11B). The map for the complete reachable workspace is not generated here. The algorithm uses a capability map based on manipulability ellipsoid, but does not encode the instrument’s additional degrees of freedom. Moreover, the computation of the map is coupled with the task definition. The setup planner provides a better-suited region for the robot base from which the physician can select a specific base position. For the chosen robot base position, the initial joint configuration of the robot can also be optimized such that it is collision-free. The authors do not specify any computational times, so a direct comparison cannot be performed.

In contrast to our approach, the method proposed in Adhami and Coste-Maniere (2003) assumes that the deployed robots have enough kinematic versatility to adapt themselves to a selected entry access point. Thus, the optimization for the entry access point is decoupled from the optimization of the robot’s base pose, and both are considered as separate problems. Moreover, the solution relies on a geometrical approach via task cone primitives to determine the visibility and dexterity of the surgical location. This does not provide an appropriate representation of the robot’s kinematic capabilities inside the patient compared to the reachability map. Furthermore, the initial robot joint configuration is optimized for a predefined target trajectory, rather than the robot base position. The overall time for the experimental results is 6 min for the entry access point optimization and 3 min for the robot joint configuration optimization.

Our proposed approach decouples the generation of the map from the task definition. The map is generated based on the kinematics of the robot, the robot base pose, and the pose of the entry access point for constrained access types. For any set of 6D task poses, the generated maps can be queried and utilized for setup planning. Moreover, since the map covers the complete workspace, it can be additionally used in the optimization cost computation, to find a robot base position with good overall reachability. RASSCMAPs can be used in preoperative task-based setup optimization for constrained and unconstrained scenarios. For instance, for a constrained surgical procedure, it aids in three different cases: 1) computing optimal robot base pose for a fixed entry access point, 2) computing optimal access entry point for a fixed robot base position, and 3) simultaneous computation of both access entry point and robot base position. Examples of these tasks and their computation times are discussed in detail in Section 5. Since RASSCMAPs for different robot base poses and access entry points are computed offline and saved in a database, they can also be easily visualized online to help the physician in intra-operative setup.

## 7 Conclusion

This work introduces a novel method for RASS setup and planning based on RASSCMAPs, i.e., capability maps (CMAPs) for RASS. The CMAPs, initially developed for robot workspace analysis, were further developed to provide surgical procedure-specific dexterity information for the robot. The RASSCMAPs can be utilized for preoperative and intra-operative setup planning. For surgeries with access constraints, for example in laparoscopy, the RASSCMAP considers the constraints imposed by the access types. The RASSCMAP provides also information for robot setup planning in surgeries with unconstrained access types.

Further development of RASSCMAP will be focused on implementing an adequate approach to include collision avoidance inside and outside the patient in multiple robot setup scenarios. Collision avoidance with prohibited areas around the robot can also be considered in the RASSCMAP generation process. Optimizing the initial joint configuration of the robot can also be considered within the scope of RASSCMAP generation by introducing other appropriate metrics in addition to the discussed reachability index.

This work also proposed a preoperative task-based setup planning, that is, location of the robot base and access points, based on genetic algorithms. The optimized robot base and access location ensure that all desired task poses are reachable with the highest possible dexterity. The proposed RASSCMAP-based setup optimization is experimentally evaluated for two use cases: pedicle screw placement with general unconstrained access type, and robot-assisted laparoscopy with constrained access type for the insertion of tools.

The setup optimization could also incorporate higher-level operative aspects such as collision avoidance for multi-arm robotic systems, using methods such as the reactive collision avoidance proposed by Dietrich et al. (2012). Furthermore, mixed reality approaches can be utilized based on the proposed RASSCMAPs, in order to aid the physicians during intra-operative system setup. Usability studies could be conducted with clinicians to further evaluate and improve the acceptance of this novel tool in surgical environments.

## Data Availability

The raw data supporting the conclusion of this article will be made available by the authors, without undue reservation.

## Footnotes

1 The description of an automated information acquisition system is not part of this paper.
